# Epiphytic Bacteria from Sweet Pepper Antagonistic In Vitro to *Ralstonia solanacearum* BD 261, a Causative Agent of Bacterial Wilt

**DOI:** 10.3390/microorganisms9091947

**Published:** 2021-09-14

**Authors:** Tshifhiwa Paris Mamphogoro, Casper Nyaradzai Kamutando, Martin Makgose Maboko, Olayinka Ayobami Aiyegoro, Olubukola Oluranti Babalola

**Affiliations:** 1 Gastro-Intestinal Microbiology and Biotechnology Unit, Agriculture Research Council-Animal Production, Private Bag X02, Irene, Pretoria 0062, South Africa; MamphogoroT@arc.agric.za; 2 Food Security and Safety Niche Area, Faculty of Natural and Agricultural Sciences, North-West University, Private Bag X2046, Mmabatho 2735, South Africa; olubukola.babalola@nwu.ac.za; 3 Department of Plant Production Sciences and Technologies, University of Zimbabwe, P.O. Box MP167, Mount Pleasant, Harare 0263, Zimbabwe; kamutandocn@gmail.com; 4 Crop Science Unit, Agriculture Research Council—Vegetable and Ornamental Plants, Private Bag X293, Roodeplaat, Pretoria 0001, South Africa; mmaboko@yahoo.com; 5 Research Unit for Environmental Sciences and Management, North-West University, Private Bag X1290, Potchefstroom 2520, South Africa

**Keywords:** antagonists, biological control, epiphytes, sweet pepper, 16S rRNA genes, *Ralstonia solanacearum*

## Abstract

Biological control of plant pathogens, particularly using microbial antagonists, is posited as the most effective, environmentally-safe, and sustainable strategy to manage plant diseases. However, the roles of antagonists in controlling bacterial wilt, a disease caused by the most devastating and widely distributed pathogen of sweet peppers (i.e., *R. solanacearum*), are poorly understood. Here, amplicon sequencing and several microbial function assays were used to depict the identities and the potential antagonistic functions of bacteria isolated from 80 red and green sweet pepper fruit samples, grown under hydroponic and open soil conditions, with some plants, fungicide-treated while others were untreated. Amplicon sequencing revealed the following bacterial strains: *Bacillus cereus* strain HRT7.7, *Enterobacter hormaechei* strain SRU4.4, *Paenibacillus polymyxa* strain SRT9.1, and *Serratia marcescens* strain SGT5.3, as potential antagonists of *R. solanacearum*. Optimization studies with different carbon and nitrogen sources revealed that maximum inhibition of the pathogen was produced at 3% (*w/v*) starch and 2,5% (*w/v*) tryptone at pH 7 and 30 °C. The mode of action exhibited by the antagonistic isolates includes the production of lytic enzymes (i.e., cellulase and protease enzymes) and siderophores, as well as solubilization of phosphate. Overall, the results demonstrated that the maximum antimicrobial activity of bacterial antagonists could only be achieved under specific environmental conditions (e.g., available carbon and nitrogen sources, pH, and temperature levels), and that bacterial antagonists can also indirectly promote crop growth and development through nutrient cycling and siderophore production.

## 1. Introduction

Sweet pepper (*Capsicum annum*), a heat-loving vegetable species, is grown worldwide, with an estimated fruit yield of more than 32.3 million tonnes, annually [1]. In a world were some communities or households can be food secure, but nutritionally insecure [2,3], peppers can bridge this gap, as they harbor important nutritional attributes. For example, risks of human disease such as cancer, heart disease, and diabetes were reported to be minimized by polyphenols and flavonoids [4,5], which are biochemicals highly concentrated in peppers [6,7,8]. Pepper fruits are usually used for spicing because of their flavour [9]. Due to these reasons, demand for sweet pepper fruits may increase, and this calls for intervention measures that can promote the productivity of this important crop on a global scale.

Yield and fruit quality of sweet peppers were previously reported to be influenced by genotype [10] as well as the farming system (i.e., agronomic management) [11]. Both, the crop genotype and crop management practices are traditionally known to affect yield development and crop quality, but this happens in an environment endowed with abiotic and biotic stress factors. For example, under disease stress, some plants may tolerate or resist infections genetically, either through physiological or biochemical mechanisms [12,13]. In order to withstand disease pressures, the plant genotype was also reported as instrumental in shaping the surrounding microbial communities, to harbour mostly those microbial taxa with plant growth-promoting potentials, including antagonists of the pathogenic taxa [14,15].

The top ten, most problematic bacterial pathogens of crops were previously listed, with *Pseudomonas syringe* pathovars and *Ralstonia solanacearum* topping the list [16]. In sweet peppers, *R. solanacearum* is regarded as the most damaging and yield constraining pathogen [17,18]. *R. solanacearum* causes a disease known as bacterial wilt. Apart from peppers, this pathogen also attacks over 200 other plant species and it is distributed worldwide, where it was observed to induce a destructive economic impact [19]. Affected plants usually show rapid and fatal wilting symptoms [20]. Over the years, farmers have struggled to control this pathogen, because of its ability to grow endophytically, survive in deep soil horizons, and migrate in water, and its ability to associate with weeds [21]. Therefore, more pragmatic approaches to control this pathogen, especially those measures that are sustainable and environmentally friendly, need to be developed.

Biological control agents (especially, antagonists) are widely accepted as sustainable and ideal for protecting the integrity of ecosystems and biodiversity [22,23,24]. For instance, field evaluations of the bacterial antagonists *Bacillus amyloliquefaciens* SQR-7 and SQR-101 and *B. methylotrophicus* SQR-29 against *R. solanacearum* showed biocontrol efficacy (BE) of 18–60% in tobacco [25]. Elsewhere, the antagonist *Ralstonia pickettii* QL-A6 indicated a BE of 73% against *R. solanacearum* in tomato plants [26]. *B. stratosphericus* strain LT743897 proved to increase secondary metabolite production in *S. lycopersicum* plants, resulting in growth promotion and in vivo antagonistic activity. The strain also stimulated the SA defense mechanism against *R. solanacearum* infecting pathogens [27]. In our recent study, 16S rRNA amplicon sequencing data of samples collected from surfaces of red and green sweet pepper fruits, grown under hydroponic (fungicide treated + untreated) and open soil (fungicide treated + untreated) systems, revealed several bacterial taxa with the potential to antagonize pathogenic microorganisms [28]. However, details on the antagonists that can suppress the most important pathogens of sweet peppers, such as *R. solanacearum*, are not yet available.

Therefore, this study aimed to isolate, characterize, and evaluate potential bacterial antagonists, residing on the surfaces of red and green sweet pepper fruits, sampled from plants grown under different management conditions (i.e., hydroponic and open soil conditions, but either fungicide-treated or untreated) for their ability to suppress *R. solanacearum* BD 261. We hypothesized that sweet pepper fruits harbor some specific bacterial strains on their surfaces that inhibit the proliferation of pathogenic strains such as *R. solanacearum* BD 261, and that these antagonists are more important on plants grown in open soil, where disease pressures are typically high.

## 2. Materials and Methods

### 2.1. Study Sites and Crop Management

Sweet peppers (cv. King Arthur) were planted in October 2014 and were maintained until March 2015, at the Agricultural Research Council-Vegetable and Ornamental Plants Institute (ARC-VOP), in Roodeplaat, Pretoria, South Africa (Latitude = 1200, Longitude = 25°59′ S; 28°35′ E). Plants were grown in a hydroponic system as well as under open field conditions. The mean temperature under hydroponic growing conditions was 33 °C day/15 °C night. In the open field, average temperatures of 34.5 °C day/15 °C night were recorded. The experimental design was a 2 (treatments, i.e., fungicide-treated (T) and untreated (U)) × 2 (growing conditions, i.e., hydroponic (H) and open field (S)) × 2 (maturity stages, i.e., green (G) and red (R) colour) factorial, with ten replicates, thereby making-up a total of 80 planting stations.

Cultivation practices of sweet pepper for the field and hydroponic growing conditions were previously reported in detail by [28]. Two weeks after transplanting (WAT), plants were treated with Copper-count (5 mL/L) Hygrotech (Pty) Ltd., Pretoria, South Africa), Sporekill (1 mL/L) (ICA International Chemicals (Pty) Ltd., Stellenbosch, South Africa), Binomyl (50 g/L) (Villa Crop Protection (Pty) Ltd., Kempton Park, South Africa), Bravo (210 mL/L) and Ridomol gold (360 mL/L) (Syngenta, Centurion, South Africa), to control against powdery mildew, blight, and leaf spot. Insecticides such as Actar (50 mL/L) (Syngenta, Centurion, South Africa), Hunter (40 mL/L) (BASF Pty Ltd., Midrand, South Africa), Diozinon (160 mL/L) (AVIMA (Pty) Ltd., Kenmare, South Africa), Biomectine (60 mL/L) (Villa Crop Protection (Pty) Ltd., Kempton Park, South Africa), and Savage (40 mL/L) (Villa Crop Protection (Pty) Ltd., Kempton Park, South Africa), were also applied to control white flies, red spider mites, and aphids.

### 2.2. Sample Collection, Processing, and Isolation of Potential Antagonists

A total of 80 (i.e., 10 HGT + 10 HRT + 10 HGU + 10 HRU + 10 SGT + 10 SRT + 10 SGU + 10 SRU), fresh, intact, and health green and red sweet pepper fruits were aseptically collected in sterile Ziploc bags and kept at 4 °C in the lab. Bacterial biofilms on the surfaces of the pepper fruits were recovered using sterile cotton swabs soaked in a solution containing 0.15M NaCl and 0.1% Tween 20, as in [29]. The swabs were vortexed in sterile Eppendorf tubes containing saline solution (0.85% NaCl). The supernatant was serially diluted and one hundred μL aliquots from the 10^−1^ and 10^−2^ dilutions were plated on Trypticase soy agar (TSA). The plates were incubated for 48 h at 30 °C under aerobic conditions. Ten colonies per plate with unique morphologies were selected based on differences in colour, shape, and texture, for further purification (i.e., n = 800, 80 swabs × 10 colonies; Table S1). Purified colonies were streaked on TSA and incubated at 37 °C for 24 h, and stored on a Trypticase soy broth (TSB) medium containing 50% glycerol at −80 °C for further use.

### 2.3. Plant Bacterial Pathogen

The plant pathogenic bacteria *R. solanacearum* BD 261 (Phylotype II race 3 biovar 2) [30], isolated from wilted tomato plants and was acquired from the culture bank of the ARC’s Plant Protection Biosystems Laboratories, in Pretoria, South Africa (www.arc.agric.za/arc-ppri). The pathogen was maintained on 2-3-5 triphenyl tetrazolium chloride (TZC) in McCartney bottles at 4 °C until use. Stock cultures of the test pathogen were prepared for use throughout the study and maintained in the culture collection of the ARC’s Gastro Intestinal Microbiology and Biotechnology Laboratories, which are under the Animal Production Institute, Irene (www.arc.agric.za/arc-api).

### 2.4. Multiplication of Potential Antagonists and the Pathogen

Multiplication of potential antagonists and pathogens followed procedures in [31], but with minor modifications. Isolates were grown on TSA medium and pathogens from TZC medium were re-cultured on sucrose peptone broth (SPB) medium containing (gL^−1^); sucrose (20), peptone (5), K_2_HPO_4_ (0.5), MgSO_4_.7H_2_O (0.25), and pH 7.2–7.4. The growth of potential antagonists and *R. solanacearum* BD 261 isolates was performed at 30 °C with shaking for 48 h [32].

### 2.5. In Vitro Screening of Isolates for Antagonism

Antibacterial activity screening of potential antagonists against *R. solanacearum* BD 261 was conducted before and after enrichment using an optimized spot-on-lawn assay [33]. Briefly, 200 μL of *R. solanacearum* BD 261 cell culture (OD_600_ ~ 0.4) was grown in SPB medium and then grown on cooled King’s B agar medium plates which contained (gL^−1^); protease peptone (20), MgSO_4_.7H_2_O (1.5), K_2_HPO_4_ (1.5), glycerol (10 mL), and agar (15), at pH 7.2. Plates were dried for 40–50 min, and five wells (5 mm in diameter) were made per plate using a cork borer, with 50 μL of each potential bacterial antagonist grown in SPB (i.e., OD_600_ ~ 0.4) was added into each well. Fifty (50) μL of cell culture of *Bacillus stratosphericus* LT743897 (OD_600_ ~ 0.4) grown in SPB was used as a positive control. The inhibition zone of the bacterial isolates on *R. solanacearum* strain BD 261 was measured after 48 h of incubation at 30 °C. The experiments were performed at least three times.

### 2.6. DNA Extraction and PCR Amplification of Potential Antagonistic Strains

#### 2.6.1. PCR Amplification of 16S rRNA Genes

Total genomic DNA of potential antagonistic strains was extracted from pure cultures using the Quick-DNA Fungal/Bacterial Miniprep Kit Zymo Research D6005 according to the manufacturer’s instructions (www.zymoresearch.com). In general, one μL of genomic DNA was used as a template to amplify the full-length of the 16S rRNA gene [34]. An approximately 1.5 kb fragment part of the 16S rRNA gene was amplified using the universal primer pair (27F:5′-AGAGTTTGATCCTGGCTCAG-3′ and 1492R:5′-GGTTACCTTGTTACGACTT-3′) [35]. Amplifications were performed in 20-μL reaction volumes containing, 10 μL One Taq 2X Master Mix with Standard Buffer (NEB, catalogue No. M048S, Invitrogen, Carlsbad, CA, USA; 1X), 1 μL of both primers: 27F and 1492R with a concentration of 10 μM, 7 μL Nuclease free water (Catalogue No. E476), and 1 μL DNA template (10–30 ng/μL).

PCR was performed using Thermal Cycler (MJ Mini Personal Thermal Cycler, Bio-Rad; www.bio-rad.com). The PCR conditions were as follows: initial denaturation at 94 °C for 3 min, followed by 30 cycles of 94 °C for 30 s, 50 °C for 30 s, 68 °C for 1:30 min, and then a final elongation step at 68 °C for 5 min. The amplified genes were ran on 1% agarose gel electrophoresis CSL-AG500 (Cleaver Scientific Ltd.; www.cleverscientific.com), stained with EZ-vision Bluelight DNA Dye with the size markers (10 kb Fast DNA ladder NEB N3238, Invitrogen, Forster city, CA, USA; www.amresco-inc.com) and then cleaned with ExoSAP, a mixture of Exonuclease I NEB M0293L and Shrimp Alkaline Phosphatase NEB M0371 (Invitrogen, Waltham, MA, USA).

#### 2.6.2. Sequencing and Bioinformatics Analysis of the 16S rRNA Amplicons

The cleaned amplicons were sequenced at Inqaba Biotechnical Industries (Pty) Limited (www.inqababiotec.co.za) in the forward and reverse direction, using the Nimagen, BrilliantDye^TM^ Terminator Cycle Sequencing Kit V3.1, BRD3 100/1000, Nijmegen, Netherlands, following the manufacturer’s instructions. Amplicons were then purified with the Zymo Research, ZR-96 DNA Sequencing Clean-up Kit D4053, Irvine, CA, USA. Purified fragments were analyzed on the ABI 3500XL Genetic Analyzer with a 50-cm array, using POP7 (Applied Biosystems, ThermoFisher Scientific., Foster City, CA, USA) for each reaction for every sample. The sequence chromatogram generated by the ABI 3500XL Genetic Analyzer were analysed using the FinchT v1.4 software, and the obtained results were compared with the related 16S-rDNA sequences identified by the Basic Local Alignment Search (BLAST) search program on the National Center for Biotechnology Information (NCBI), National Library of Medicine, USA [36].

Sequence alignments were performed using the CLUSTLW algorithm in MEGA v6.06 [37] with default settings, and phylogenetic trees were constructed using the neighbor-joining method [38]. Reliability of the phylogenetic tree was evaluated through bootstrap analysis with 1000 re-samplings using a p-distance model, with the numbers on branches indicating percentage level of bootstrap support as described in [38].

#### 2.6.3. Nucleotide Sequence Accession Numbers

The nucleotide sequences of the 16S rRNA genes has been deposited in the GenBank database under accession numbers MN911398.1—MN911401.1.

### 2.7. Optimization for Improved Activity of Potential Antagonistic Strains

The screened bacterial antagonistic strains were inoculated into SPB enriched with different compositions of carbon sources (including; fructose, glucose, lactose, maltose and starch) and nitrogen sources (i.e., ammonium sulfate, ammonium chloride glycine, yeast and tryptone) at different pHs (ranging from 5–9, adjusted with 1N HCl and 1N NaOH), and incubated at 30 °C on shaker for 48 h as in [27,39]. After incubation, the antagonistic strains were screened against plant pathogens *R. solanacearum* BD 261 on King’s B agar plates using a spot-on-lawn assay at different pHs and observed for inhibition zones as described in [33]. The antagonists were then cultured at concentrations of 0.5, 1, 1.5, 2, 2.5, and 3% (*w/v*) with optimized carbon and nitrogen sources and pH. Potent strains displaying the highest potential for *R. solanacearum* BD 261 suppression at the highest concentration of optimized carbon, nitrogen sources, and pH were allowed to grow at 25, 28, 30, 35, and 37 °C for 24–48 h together with *R. solanacearum* BD 261, using the perforated agar plate technique [32]. Plates were then examined for inhibition zones which were measured and recorded. The temperature exhibiting the maximum suppression of *R. solanacearum* BD 261 was recognized as the optimum temperature for determining antagonistic effects of the pathogen for further studies. The experiments were performed at least three times.

### 2.8. Determination of Potential Antimicrobial Traits

#### 2.8.1. Cellulase Activity

Cellulase activity was determined as described by [40] with minor modifications. Briefly, the supernatants of antagonistic strains (50 μL) were inoculated into the wells of carboxymethyl cellulose (CMC) agar medium containing (gL^−1^): KH_2_PO_4_ (1.0), MgSO_4_·7H_2_O (0.5), NaCl (0.5), FeSO_4_·7H_2_O (0.01), MnSO_4_·H_2_O (0.01), NH_4_NO_3_ (0.3), CMC (10) and agar (15). After incubation at 25 °C for 72 h, plates were flooded with 0.1% Congo red for 20 min and then with 1M NaCl for 20 min. Production of cellulase was identified by a zone hydrolysis formation around the colonies. The experiment was conducted in triplicate.

#### 2.8.2. Protease Activity

Protease activity was determined by inoculating antagonistic strains. Supernatants (50 μL) were added into wells of LB agar medium containing 3% skim milk powder and incubated at 28 °C for 72 h. A clear zone around the test strains after incubation was used as an indicator for protease production [41]. The experiment was conducted in triplicate.

#### 2.8.3. Detection of Phosphate Solubilization

Phosphate solubilization was carried out in a minimal medium, according to [42] with slight modification. This medium contained (gL^−1^): glucose (10), Ca_3_(PO4)_2_ (5), (NH_4_)_2_SO_4_ (0.5), NaCl (0.2), MgSO_4_·7H_2_O (0.1), KCl (0.1), yeast extract (0.2), MnSO_4_·H_2_O (0.001), FeSO_4_·7H_2_O (0.001) and agar (20) at pH 6.8. After cooling the media to 50 °C, supernatants of the antagonistic strains (50 μL) were added on the wells of the medium plates and incubated for 72 h at 25 °C. Phosphate solubilization was determined by observing a clear zone around the colonies. The experiment was conducted in triplicate.

#### 2.8.4. Siderophore Production

Production of siderophores was assessed by the universal modified chemical assay using Chrome azurol S (CAS) agar medium prepared as in [43]. The CAS agar plates were used to detect for presence of siderophores in culture supernatants of the potential antagonistic strains. The CAS agar plates consist of two main components (i.e., the CAS indicator solution and the Basal agar medium). The CAS indicator solution was prepared by dissolving 60.5 mg CAS in 50 mL distilled water, mixed with 10 mL of Fe^+3^ (27 mg FeCl_3_·6H_2_O, and 83 mL conc. HCl in 100 mL ddH_2_O). Additionally, 72.9 mg hexadecyltrimethylammonium bromide (HDTMA) dissolved in 40 mL distilled water was also slowly added while stirring to give a dark blue 100 mL total volume. The solution was autoclaved before use.

The Basal agar medium consisted of a mixture of 10 mL MM9 salt stock solution which contained 30 g KH_2_PO_4_, 50 g NaCl and 100 g NH_4_Cl in 1 L ddH_2_O, 3.23 g PIPES and 12 g of NaOH, all dissolved in 75 mL using distilled water, with pH adjusted to 6.8. After adjusting the pH, 1.2 g agar was added while stirring. The resultant solution was then autoclaved. After cooling the media to 50 °C, 10 mL blue dye solution, 3 mL of 10% Casamino acid solution, and 10 mL of 20% glucose as a carbon source were slowly added along the glass wall with adequate agitation to blend thoroughly. The potential antagonistic strains supernatants (50 μL) were applied in a well on each CAS plate, and the plates were incubated at 25 °C for 72 h. Observation of formation of yellow-orange halos around the bacterial colonies designated siderophore production. The experiment was conducted in triplicate.

### 2.9. Statistical Data Processing

Antibacterial activity screening (i.e., inhibition zones) collected before and after enrichment and gathered data (i.e., inhibition zones) for each of the different treatment levels (i.e., pH, carbon and nitrogen sources, temperature, etc.) was subjected, firstly, to analysis of variance (ANOVA) using the ‘*aov*’ function in the agricolae v1.3-1 R package. Statistical differences between the isolates and the positive control in suppressing the *R. solanacearum* strain BD 261 were detected using the Tukey’s HSD test, using the ‘*TukeyHSD*’ function in the agricolae R package [44]. In order to visualize how the isolates differ in performance (i.e., pathogen suppression), in comparison with the control isolate, a scatter plot was used. Scatter plots were graphed using the ‘*ggplot*’ function in the ggplot2 v3.0.0 R package [45].

## 3. Results

### 3.1. Isolation and Identification of Potent Bacterial Strains

Bacterial isolations yielded a total of 800 colonies (i.e., isolates) that showed unique morphologies in terms of colour, shape, and texture. In particular, the 800 isolates consisted of 10 colonies selected from each of the 80 sweet pepper fruit samples (i.e., 10 HGT (hydroponic green treated) + 10 HRT (hydroponic red treated) + 10 HGU (hydroponic green untreated) + 10 HRU (hydroponic red untreated) + 10 SGT (soil green treated) + 10 SRT (soil red treated) + 10 SGU (soil green untreated) + 10 SRU (soil red untreated)) (Table S1). Among the 800 isolated strains, only four exhibited inhibitory effects against *R. solanacearum* BD 261. These antagonistic strains were identified as HRT7.7, SGT5.3, SRT9.1, and SRU4.4 (Table S1; Figure S1).

**Figure 1:**
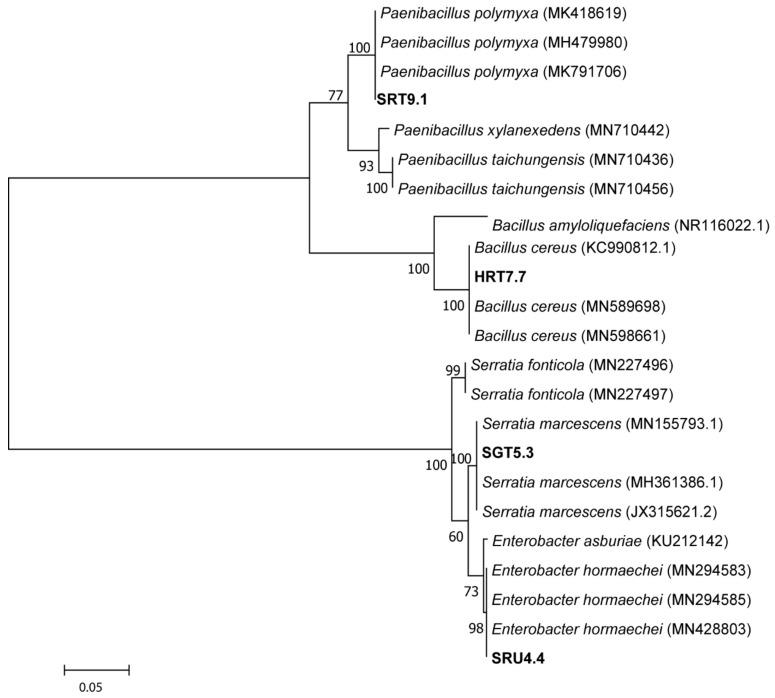
Neighbor-joining phylogenetic tree based on 16S rRNA gene sequences of potential antagonistic strains showing the relationship of closest type strain sequences. The phylogenetic tree was constructed using the neighbour-joining algorithm. The tree is based on 1000 resampled datasets and the numbers on branches indicate the percentage level of bootstrap support.

Briefly, the isolate identified as HRT7.7 showed the highest sequence similarity with *Bacillus cereus* MN589698 (99%), SRU4.4 shared the highest sequence similarity with *Enterobacter hormaechei* MN428803 (98%), SRT9.1 showed close identity with *Paenibacillus polymyxa* MK791706 (99%), while SGT5.3 was similar to *Serratia marcescens* MN155793.1 (99%). Based on these similarities, the isolates have been coded as below: HRT7.7 as *Bacillus cereus* strain HRT7.7; SRT9.1 as *Paenibacillus polymyxa* strain SRT9.1; SGT5.3 as *Serratia marcescens* strain SGT5.3; and lastly, SRU4.4 as *Enterobacter hormaechei* strain SRU4.4 (Table 1). Phylogenetic analysis of the 16S rRNA gene sequences showed that the isolated antagonistic strains clustered with other genera of *Bacillus*, *Enterobacter*, *Paenibacillus,* and *Serratia* (Figure 1).

**Figure 2:**
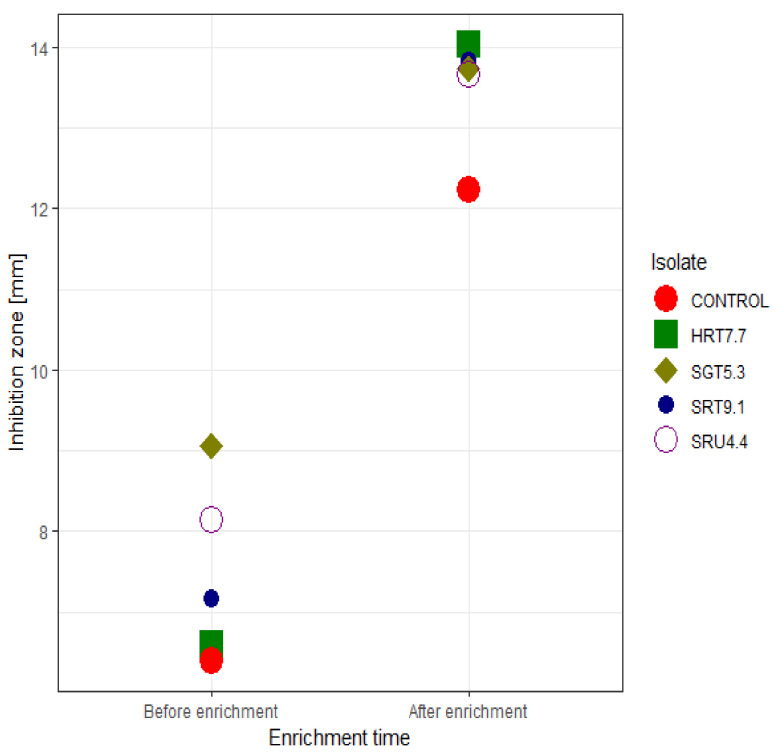
A scatter plot showing inhibition zones of the sweet pepper fruits isolates against the *R. solanacearum* BD 261 pathogenic strain, before and after enrichment.

Assessing the antagonistic potential of the isolates, before and after enrichment, also revealed some interesting trends. Firstly, the four isolates and the control strain significantly differed (*p* < 0.05) in their ability to suppress the *R. solanacearum* BD 261 strain, both before and after enrichment (Table S2). Generally, before enrichment, all the isolates (including the control) showed low potential in inhibiting the pathogenic strain. However, the strains, SRT9.1 (*Paenibacillus polymyxa* strain SRT9.1) and SRU4.4 (*Enterobacter hormaechei* strain SRU4.4), with inhibition zones of 8.1 mm and 9.1 mm, respectively, exhibited a huge potential in suppressing the pathogenic strain before enrichment. After enrichment, a jump in antagonistic potential was shown for all the isolates, together with the control (Figure 2; Table S3). Interestingly, the inhibitory potential of the control was significantly (*p* < 0.05) lower than all of the newly identified antagonistic strains (Table S4).

### 3.2. Optimization for Enhanced Antagonistic Activity

**Figure 3:**
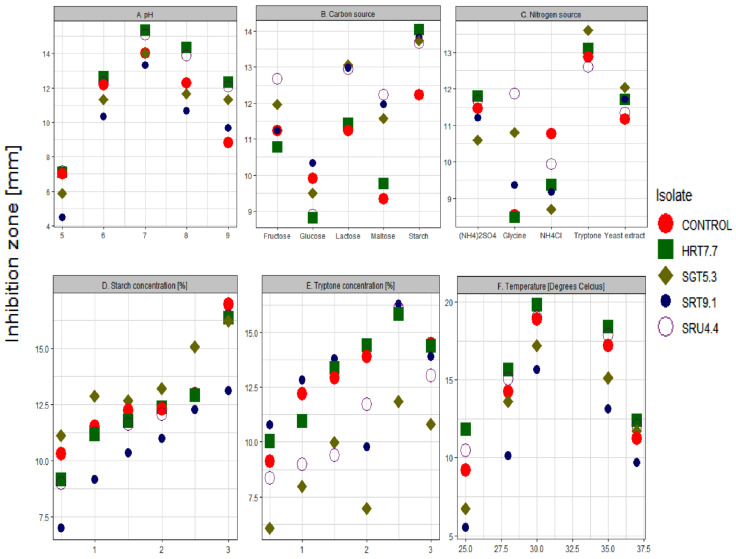
A scatter plot showing inhibition zones of the sweet pepper fruits isolates against the *R. solanacearum* BD 261 pathogenic strain, at different treatment levels of (**A**) pH, (**B**) carbon sources, (**C**) nitrogen sources, (**D**) starch concentrations, (**E**) tryptone concentrations, and (**F**) temperature.

Determining the effects of the different treatment levels of pH, carbon and nitrogen source, temperature, and the concentration of carbon and nitrogen source (starch and tryptone) on the antagonistic potential of the bacterial isolates from the sweet pepper fruit samples showed encouraging results. First, at these different treatment levels, the isolates differed significantly (i.e., *p* < 0.05) in their ability to deter the function of the pathogenic strain *R. solanacearum* BD 261 (except for pH = 6 and the yeast extract treatments; Table 2). The highest antagonistic activity was observed at a neutral pH, but pH levels above 6, all seemed to enhance the inhibitory activities of the antagonistic strains, with inhibitory zones above 10 mm, in most cases (Figure 3A; Table S5). Furthermore, the effects of the isolates at different pH significantly differed from the control (Table S6).

Carbon sources, including lactose, fructose, and starch, influenced the antagonistic potential of the isolates to the highest degree. Starch proved to be the ideal carbon source, with inhibition zones above 13.5 mm for all the isolates (Figure 3B; Table S5). Of note, all the isolates significantly differed from the control in their ability to inhibit the pathogenic strain when supplied with starch (Table S6). Additionally, the antagonists seemed to favour starch at higher concentrations for optimal activity (Figure 3D).

Although the nitrogen sources (NH_4_)_2_SO_4_, yeast extract, and tryptone revealed an immense potential in the aiding activity of the antagonists against the pathogenic strain, *R. solanacearum* BD 261, tryptone was observed as the ideal nitrogen source (Figure 3C). Inhibitory zones of the isolates, together with the control, were all greater than 12.5 mm for the tryptone treatment and greater than those observed for the other nitrogen sources (Table S5). For this treatment, no meaningful differences in activity were detected between the isolates and the control (Table S6). However, as observed for tryptone, it is important to note that the activity of the isolates, under an environment enriched with tryptone, tended to be much higher at higher concentration levels (Figure 3E). Lastly, temperatures ranging from 27–35 °C were observed as ideal for promoting the activity of the antagonists against the pathogenic strain. However, the maximum activity was observed at a temperature of 30 °C (Figure 3F).

### 3.3. Determination of Antimicrobial Traits of the Antagonists

**Figure 4:**
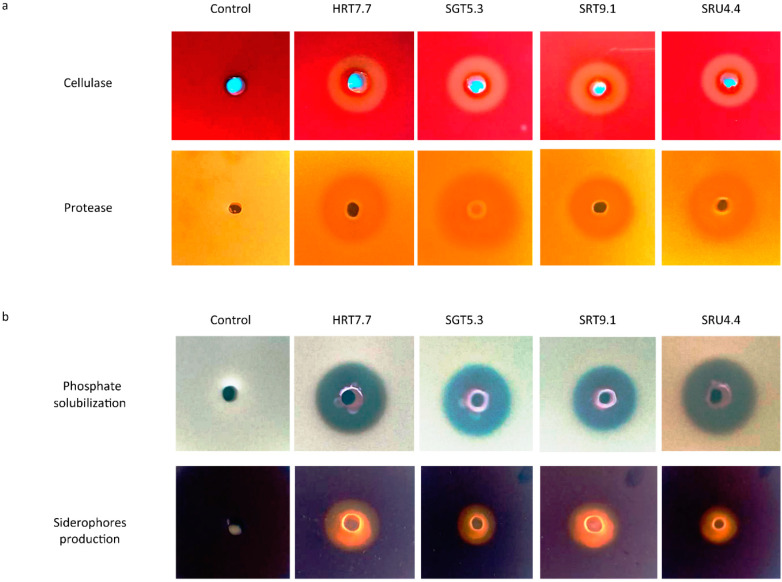
Production of antimicrobial traits by *Bacillus cereus* (HRT7.7), *Paenibacillus polymyxa* (SRT9.1), *Serratia marcescens* (SGT5.3), and *Enterobacter hormaechei* (SRU4.4). (**a**) Production of cellulase and protease, (**b**) phosphate solubilization, and siderophore production.

*Bacillus cereus* (HRT7.7), *Paenibacillus polymyxa* (SRT9.1), *Serratia marcescens* (SGT5.3), and *Enterobacter hormaechei* (SRU4.4) were evaluated for secondary metabolite production associated with antimicrobial activity, including cellulase and protease, on LB plates containing, CMC, and skim milk. Clear zones around the isolates exhibited their high cellulase and proteolytic activity (Figure 4a). Additionally, solubilization of insoluble phosphate and siderophore production were also depicted by the clear zone halos around wells containing colonies and the yellow-orange halos formation around the CAS agar plates (Figure 4b). The assays clearly showed that the isolates potentially antagonize *R. solanacearum* using lytic enzymes and siderophore production, as well as by solubilizing phosphate, as their mode of action (Table S7).

## 4. Discussion

Biological control (particularly, using antagonists) is poised as the most sustainable and environmentally safe, disease control strategy in crop production [22,23,24]. However, the roles of antagonists in controlling bacterial wilt, a disease caused by the most devastating and widely distributed pathogen of sweet peppers (i.e., *R. solanacearum*), are poorly understood. Here, potential bacterial antagonists were isolated from 80 red and green sweet pepper fruit samples, grown under hydroponic and open soil conditions, with some plants fungicide-treated while others were untreated. Amplicon sequencing of 16S rDNA of the identified potential antagonists, together with microbial activity assays, showed the identities of the isolates as potential antagonists against *R. solanacearum* and revealed the optimal conditions of activity, as well as the mode of action of the isolates against the pathogenic strains.

Firstly, identification of the isolates *Bacillus cereus* strain HRT7.7, *Paenibacillus polymyxa* strain SRT9.1, *Serratia marcescens* strain SGT5.3, and *Enterobacter hormaechei* strain SRU4.4, as antagonists of *R. solanacearum*, was not surprising since several strains in the genera *Bacillus*, *Enterobacter*, *Serratia,* and *Paenibacillus* were previously reported to suppress *R. solanacearum* in vitro [46]. The ability of these strains to inhibit the growth of phytopathogenic bacteria, as observed in this study, places them as suitable biocontrol agents in crop production.

Different studies have demonstrated that temperature is one of the significant factors that influence microbial antagonist growth and activity [47,48]. Our results also demonstrated temperature as an essential parameter in determining the antagonistic activity of bacterial antagonists against *R. solanacearum* BD 261. Although temperatures that range between 27–35 °C were ideal for the antagonistic activity, 30 °C was optimal. This finding has several implications for decision-making in crop production. For instance, since the antagonists prefer on average high temperatures for maximum activity, this suggests that application of the bacteria as a biological control measure on the crop should be made in the afternoon when temperatures are high. However, for horticultural crops such as sweet peppers, which are predominantly grown under controlled environments (e.g., greenhouses), after applying these antagonists, it would be good to maintain temperatures at 30 °C (i.e., the optimal temperature), in order to encourage maximum suppression of the pathogen. These high temperatures will not affect the sweet pepper plants physiologically since the plants are thermophilic in nature [1].

As in agreement with [49], the present results exhibited the antagonistic activity against *R. solanacearum* over a wide pH range (Figure 3A), with the maximum antimicrobial activity at pH 7. At an optimal pH level, cell growth and enzyme production (e.g., lytic enzymes) occur [49]. Several previous studies reported that near-neutral pH is appropriate for most bacteria to synthesize antagonistic substances [50].

Apart from supporting microbial growth, the amendment of the medium with carbon and nitrogen sources is known to strongly influence antimicrobial activity and synthesis of antimicrobial metabolites by microbial strains [51]. The present study depicted that carbon and nitrogen sources (particularly, high concentration of starch and tryptone) in the growth medium play an important role in encouraging antagonistic activity against *R. solanacearum* BD 261 (Figure 3). Interestingly, these results strongly agreed with previous studies, in which antimicrobial activity of *B. cereus* [52], *E. hormaechei* [53], *P. polymyxa* [54], and *S. marcescens* [55] were shown to be strongly influenced by the medium with carbon and nitrogen sources. These findings could as well help agro-chemical companies that will be interested in packaging these potential antagonists as bio-control pesticides. For instance, in formulations, antagonistic bacteria can be mixed with the most important carbon and nitrogen sources identified in this study (i.e., starch and tryptone), as this will improve the efficacy of these bio-pesticides against the *R. solanacearum* BD 261 pathogen.

Previous studies by [51,56] reported that several antagonistic bacteria (e.g., *Bacillus* spp., *Paenibacillus* spp., *Serratia* spp. and other *Enterobacter* spp.) secrete lytic enzymes including amylase, cellulases, and chitinases, which are capable of degrading chitin. The secretion of these enzymes is considered as the major and the most effective antagonistic mode of action deployed by various bacteria against plant phytopathogens [57]. Apart from suppressing pathogenic microbes, antagonists also indirectly promote plant growth and development through organic matter decomposition, phosphate solubilization, and siderophore production [58]. The present results corroborate these previous accessions, as siderophores and phosphate solubilization potential was also shown (Figure 4b). In addition, cellulase and protease activity depicted by the isolates against *R. solanacearum* was also reported in previous studies [59,60]. To the best of our knowledge, this is the first report of the isolation of a comprehensive range of epiphytic bacteria with antagonistic potential, from the surface of a fruit crop.

## 5. Conclusions

In conclusion, we have successfully isolated effective antagonistic strains from the surfaces of fresh red and green sweet pepper fruits viz. *Bacillus cereus* strain HRT7.7, *Paenibacillus polymyxa* strain SRT9.1, *Serratia marcescens* strain SGT5.3, and *Enterobacter hormaechei* strain SRU4.4. These strains exhibited a strong antagonistic activity for suppressing *R. solanacearum* in vitro, by secreting lytic enzymes such as cellulase and protease. The strains further exhibited the capability of solubilizing phosphate and siderophores production, making them good candidates as biocontrol and noble plant growth-promoting (PGP) agents. As in vitro studies should be considered before the commencement of any green house and field studies, the present study delivers a piece of convincing evidence that surface fresh pepper fruits (especially, from plants grown under open soil environments) harbor bacteria with the ability to offer plant protection against phytopathogens. Future investigation of these beneficial strains will involve the analysis of the expression of defense-related genes such as phenylalanine ammonia lyase in pepper plants and an evaluation of their ability to control *R. solanacearum* BD 261 and other pathogens in vivo, under different environmental conditions and cultural practices. In order to understand the pathways and mechanisms of suppressing the pathogen, further studies will encompass analyzing antagonist strains whole-genome sequencing. Additionally, in the future, the establishment of the relationship between metabolite or antioxidant production by the sweet pepper fruits treated with these antagonistic strains and the level (i.e., growth and antibacterial activity) will be of paramount importance since all plants deploy inherent mechanisms to resist or tolerate both the abiotic and biotic stresses.

## Figures and Tables

**Table 1:** Molecular identification of 16S rRNA gene of epiphytic bacterial strains with in vitro antagonistic traits.

Sample No	Strain Code ^a^	Base Pair Length ^b^	Species Name	Accession No ^c^	Similarity (%)
1	HRT7.7	1264 bp	*Bacillus cereus* strain HRT7.7	MN911398.1	99
2	SGT5.3	1254 bp	*Serratia marcescens* strain SGT5.3	MN911401.1	99
3	SRT9.1	1265 bp	*Paenibacillus polymyxa* strain SRT9.1	MN911399.1	99
4	SRU4.4	1255 bp	*Enterobacter hormaechei* strain SRU4.4	MN911400.1	98

^a^ Code for the selected strains with antagonistic traits. ^b^ Fragment length of selected strain. ^c^ GeneBank sequence accession numbers of selected strains.

**Table 2:** Analysis of variance (ANOVA) for antagonistic activity of the sweet pepper fruit isolates against the *R. solanacearum* BD 261 pathogenic strain, at different treatment levels of pH, carbon sources and nitrogen sources, temperature, and different level of carbon and nitrogen sources (starch and tryptone).

		pH						Carbon Sources					
Source of variation	Degrees of freedom	5	6	7	8	9	─	Glucose	Starch	Lactose	Maltose	Fructose	─
Replication	2	0.002	0.42467	0.32067	0.9613	0.4687	─	0.26467	0.162	0.05067	0.2167	0.42467	─
Treatment	4	4.071 ***	2.65	2.053 *	27.907 ***	7.036 ***	─	1.278 **	1.562 **	2.489 ***	5.304 ***	1.674 **	─
Residual error	8	0.032	0.733	0.35317	1.4853	0.2845	─	0.14967	0.14367	0.114	0.1525	0.2005	─
		**Nitrogen source**						**Temperature (°C)**						
	Degrees of freedom	Glycine	Yeast extract	Tryptone	(NH_4_)_2_SO_4_	NH_4_CL	─	25	28	30	35	37	─
Replication	2	0.1147	0.14467	0.042	0.15267	0.66467	─	0.1647	0.3247	0.1167	0.126	0.2847	─
Treatment	4	6.631 ***	0.34933	0.413 *	0.676 *	1.893 *	─	20.464 ***	14.142 ***	8.259 ***	14.451 ***	3.424 **	─
Residual error	8	0.1672	0.39883	0.08867	0.161	0.38217	─	0.2738	0.3163	0.0775	0.0677	0.3388	─
		**Starch concentration (%)**						**Tryptone concentration (%)**						
	Degrees of freedom	0.5	1	1.5	2	2.5	3	0.5	1	1.5	2	2.5	3
Replication	2	0.0347	0.206	0.006	0.08867	0.0107	0.1607	0.0127	0.026	0.0507	0.1047	0.3227	0.1847
Treatment	4	7.367 ***	5.353 ***	2.297 ***	1.922 ***	3.383 ***	7.034 **	9.837 ***	12.944 ***	12.612 ***	28.236 ***	10.561 ***	6.884 ***
Residual error	8	0.0563	0.0693	0.05933	0.04783	0.0557	0.544	0.1552	0.0443	0.1498	0.1755	0.2652	0.1563

* = 0.05 significant variation. ** = 0.01 significant variation. *** = 0.001 significant variation.

## Data Availability

The partial sequenced data (16S rRNA region) is available at the GenBank database with accession number, MN911398.1—MN911401.1. (https://www.ncbi.nlm.nih.gov/nuccore/MN911398.1; https://www.ncbi.nlm.nih.gov/nuccore/MN911401.1; https://www.ncbi.nlm.nih.gov/nuccore/MN911399.1; https://www.ncbi.nlm.nih.gov/nuccore/MN911400.1; accessed on 9 January 2020).

## Supplementary Materials

The following are available online at https://www.mdpi.com/article/10.3390/microorganisms9091947/s1, Figure S1: Antagonistic activity of HRT7.7, SGT5.3, SRT9.1, SRU4.4 and Bacillus stratosphericus (LT743897) positive control against Ralstonia solanacearum pathogen, Figure S2: Agarose gel electrophoresis analysis of 16S rRNA genes amplified from four unknown bacterial isolates using primers 27F/1492R. PCR amplified products were run on 1% agarose gel. Lane M contains the DNA Ladder (NEB Fast DNA Ladder Mix 0.5 kb–10 kb, catalogue number N3238S), lane 1: HRT7.7, lane 2: SGT5.3, lane 3: SRT9.1, lane 4: SRU4.4. Table S1: The 800 morphologically distinct colonies isolated from the 80 green and red sweet pepper fruit samples grown under hydroponic and open soil conditions (fungicide-treated and untreated) at the ARC-Vegetables and Ornamental Center in South Africa, during the 2014–2015 autumn and summer season (supplied as an excel sheet), where negative means incapable of suppressing the pathogen and positive means capable of suppressing the pathogen. Table S2: Analysis of variance (ANOVA) for bacterial colonies with potential antagonistic effects, isolated from sweet pepper fruit surfaces, against the R. solanacearum BD 261 pathogenic strain, before and after enrichment. Table S3: Antagonistic potential of bacterial isolates from green and red sweet pepper fruit samples, grown under hydroponic and open soil conditions (but, either fungicide-treated or untreated) at the ARC-VOC, during the 2014–2015 autumn and summer season in South Africa, against the R. solanacearum BD 261 strain, before and after enrichment. Table S4: Turkey’s HSD mean comparisons of the bacterial isolates from green and red sweet pepper fruit samples, grown under hydroponic and open soil conditions (but, either fungicide-treated or untreated) at the ARC-VOC, during the 2014–2015 autumn and summer season in South Africa, against the R. solanacearum BD 261 strain, before and after enrichment. Table S5: Antagonistic activity of sweet pepper fruit isolates, against the R. solanacearum BD 261 strain, at different treatment levels of pH, carbon sources and nitrogen sources, temperature, starch and tryptone. Table S6: Turkey’s HSD mean comparisons of antagonistic activity of the sweet pepper fruit isolates, against the R. solanacearum BD 261 strain, at different treatment levels of pH, carbon sources and nitrogen sources, temperature, different level of carbon and nitrogen sources (starch and tryptone) (supplied as an excel sheet). Table S7: Specific modes of action by antagonistic bacteria against R. solanacearum BD 261.
