# Supplementary material for: Epiphytic Bacteria from Sweet Pepper Antagonistic In Vitro to Ralstonia solanacearum BD 261, a Causative Agent of Bacterial Wilt
Source: Microorganisms. 2021 Sep 14;9(9):1947. doi: 10.3390/microorganisms9091947 (PMC8469110; doi:10.3390/microorganisms9091947)
Supplement: Supplementary file 1 [file microorganisms-09-01947-s001.zip › microorganisms-1340243-supplementary.pdf]

Epiphytic bacteria from sweet pepper antagonistic *in vitro* to *Ralstonia solanacearum* BD 261, a causative agent of bacterial wilt

Tshifhiwa Paris Mamphogoro[1, 2], Casper Nyaradzai Kamutando[3], Martin Makgose Maboko[4], Olayinka Ayobami Aiyegoro[1,5*], Olubukola Oluranti Babalola[2]

Supplementary Material

Figure S1 Antagonistic activity of HRT7.7, SGT5.3, SRT9.1, SRU4.4 and *Bacillus stratosphericus* (LT743897) positive control against *Ralstonia solanacearum* pathogen.

Figure S2 Agarose gel electrophoresis analysis of 16S rRNA genes amplified from four unknown bacterial isolates using primers 27F/1492R. PCR amplified products were run on 1% agarose gel. Lane M contains the DNA Ladder (NEB Fast DNA Ladder Mix 0.5 kb – 10 kb, catalogue number N3238S), lane 1: HRT7.7, lane 2: SGT5.3, lane 3: SRT9.1, lane 4: SRU4.4.

Table S1 The 800 morphologically distinct colonies isolated from the 80 green and red sweet pepper fruit samples grown under hydroponic and open soil conditions (fungicide-treated and untreated) at the ARC-Vegetables and Ornamental Center in South Africa, during the 2014-15 autumn and summer season (supplied as an excel sheet), where negative means incapable of suppressing the pathogen and positive means capable of suppressing the pathogen.

Table S2 Analysis of variance (ANOVA) for bacterial colonies with potential antagonistic effects, isolated from sweet pepper fruit surfaces, against the *R. solanacearum* BD 261 pathogenic strain, before and after enrichment.

Table S3 Antagonistic potential of bacterial isolates from green and red sweet pepper fruit samples, grown under hydroponic and open soil conditions (but, either fungicide-treated or untreated) at the ARC-VOC, during the 2014-15 autumn and summer season in South Africa, against the *R. solanacearum* BD 261 strain, before and after enrichment.

Table S4 Turkey's HSD mean comparisons of the bacterial isolates from green and red sweet pepper fruit samples, grown under hydroponic and open soil conditions (but, either fungicide-treated or untreated) at the ARC-VOC, during the 2014-15 autumn and summer season in South Africa, against the *R. solanacearum* BD 261 strain, before and after enrichment.

Table S5 Antagonistic activity of sweet pepper fruit isolates, against the *R. solanacearum* BD 261 strain, at different treatment levels of pH, carbon sources and nitrogen sources, temperature, starch and tryptone.

Table S6 Turkey's HSD mean comparisons of antagonistic activity of the sweet pepper fruit isolates, against the *R. solanacearum* BD 261 strain, at different treatment levels of pH, carbon sources and nitrogen sources, temperature, different level of carbon and nitrogen sources (starch and tryptone) (supplied as an excel sheet).

Table S7 Specific modes of action by antagonistic bacteria against *R. solanacearum* BD 261.

Figure S1

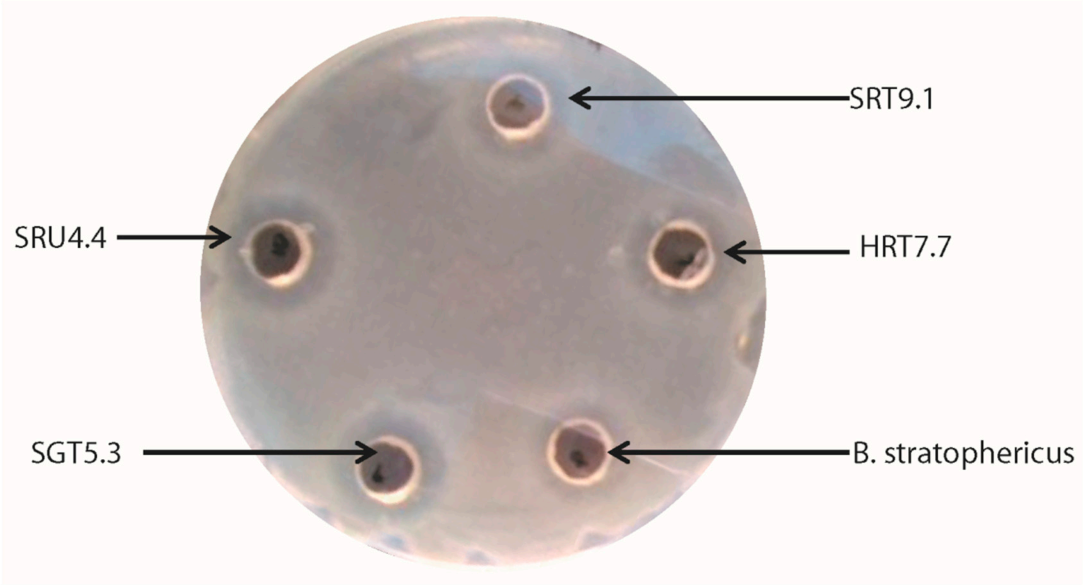

Figure S2

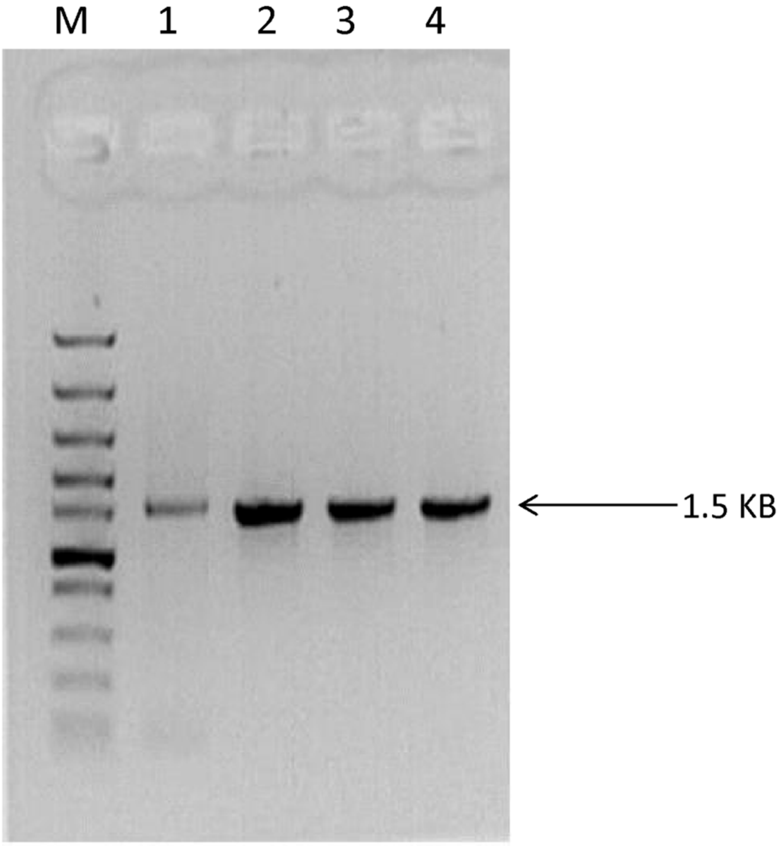

Table S2 Analysis of variance (ANOVA) for bacterial colonies with potential antagonistic effects.

		Before enrichment				After enrichment			
Source of variation	Degrees of freedom	Sum of Squares (SS)	Mean of Squares (MS)	F-value	P-value	SS	MS	F-value	P-value
Replication	2	0.105	0.053	0.471	0.641	0.324	0.162	1.1276	0.370322
Treatment	4	14.949	3.737	33.419	4.85E-05	6.2467	1.56167	10.8701	0.002553
Residuals	8	0.895	0.112			1.1493	0.14367		

Table S3 Antagonistic potential of bacterial isolates from green and red sweet pepper fruit samples, grown under hydroponic and open soil conditions (but, either fungicide-treated or untreated).

Isolate	Treatment	Inhibition zone [mm]
SGT5.3	Before enrichment	9.066667
SRU4.4	Before enrichment	8.133333
SRT9.1	Before enrichment	7.166667
HRT7.7	Before enrichment	6.6
CONTROL	Before enrichment	6.4
HRT7.7	After enrichment	14.03333
SRT9.1	After enrichment	13.83333
SGT5.3	After enrichment	13.73333
SRU4.4	After enrichment	13.66667
CONTROL	After enrichment	12.23333

Table S4 Turkey's HSD mean comparisons of the bacterial isolates from green and red sweet pepper fruit samples, grown under hydroponic and open soil conditions (but, either fungicide-treated or untreated).

**Treatment**	**Comparisons**	**Difference**	**Lower**	**Upper**	**P-value**
Before enrichment	HRT7.7-CONTROL	0.2	-0.64975	1.049754	0.9323273
	SGT5.3-CONTROL	2.666667	1.816913	3.51642	0.0000091
	SRT9.1-CONTROL	0.766667	-0.08309	1.61642	0.0822164
	SRU4.4-CONTROL	1.733333	0.88358	2.583087	0.0003929
	SGT5.3-HRT7.7	2.466667	1.616913	3.31642	0.0000186
	SRT9.1-HRT7.7	0.566667	-0.28309	1.41642	0.2563173
	SRU4.4-HRT7.7	1.533333	0.68358	2.383087	0.0010483
	SRT9.1-SGT5.3	-1.9	-2.74975	-1.05025	0.0001829
	SRU4.4-SGT5.3	-0.93333	-1.78309	-0.08358	0.0302507
	SRU4.4-SRT9.1	0.966667	0.116913	1.81642	0.0247731
After enrichment	HRT7.7-CONTROL	1.8	0.768561	2.831439	0.0013578
	SGT5.3-CONTROL	1.5	0.468561	2.531439	0.0051652
	SRT9.1-CONTROL	1.6	0.568561	2.631439	0.0032684
	SRU4.4-CONTROL	1.433333	0.401894	2.464773	0.0070532
	SGT5.3-HRT7.7	-0.3	-1.33144	0.731439	0.8678983
	SRT9.1-HRT7.7	-0.2	-1.23144	0.831439	0.9650678
	SRU4.4-HRT7.7	-0.36667	-1.39811	0.664773	0.7675204
	SRT9.1-SGT5.3	0.1	-0.93144	1.131439	0.9973615
	SRU4.4-SGT5.3	-0.06667	-1.09811	0.964773	0.9994595
	SRU4.4-SRT9.1	-0.16667	-1.19811	0.864773	0.9818279

Table S5 Antagonistic activity of sweet pepper fruit isolates, against the *R. solanacearum* BD 261 strain, at different treatment levels of pH, carbon sources and nitrogen sources, temperature, different level of carbon and nitrogen sources (starch and tryptone)

Treatment	Treatment level	Isolate	Inhibition zone [mm]
pH	5	SRU4.4	7.166667
pH	5	HRT7.7	7.1
pH	5	CONTROL	7
pH	5	SGT5.3	5.866667
pH	5	SRT9.1	4.466667
pH	6	HRT7.7	12.66667
pH	6	SRU4.4	12.33333
pH	6	CONTROL	12.16667
pH	6	SGT5.3	11.33333
pH	6	SRT9.1	10.33333
pH	7	HRT7.7	15.33333
pH	7	SRU4.4	15.06667
pH	7	CONTROL	14.03333
pH	7	SGT5.3	14
pH	7	SRT9.1	13.33333
pH	8	HRT7.7	14.33333
pH	8	SRU4.4	13.86667
pH	8	CONTROL	12.3
pH	8	SGT5.3	11.66667
pH	8	SRT9.1	10.66667
pH	9	HRT7.7	12.33333
pH	9	SRU4.4	12.06667
pH	9	SGT5.3	11.33333
pH	9	SRT9.1	9.666667
pH	9	CONTROL	8.833333
Carbon source	Glucose	SRT9.1	10.33333
Carbon source	Glucose	CONTROL	9.9
Carbon source	Glucose	SGT5.3	9.5
Carbon source	Glucose	SRU4.4	8.9
Carbon source	Glucose	HRT7.7	8.8
Carbon source	Starch	HRT7.7	14.03333
Carbon source	Starch	SRT9.1	13.83333
Carbon source	Starch	SGT5.3	13.73333
Carbon source	Starch	SRU4.4	13.66667
Carbon source	Starch	CONTROL	12.23333
Carbon source	Lactose	SGT5.3	13.06667
Carbon source	Lactose	SRT9.1	12.96667
Carbon source	Lactose	SRU4.4	12.93333
Carbon source	Lactose	HRT7.7	11.43333
Carbon source	Lactose	CONTROL	11.23333
Carbon source	Maltose	SRU4.4	12.23333
Carbon source	Maltose	SRT9.1	11.96667
Carbon source	Maltose	SGT5.3	11.56667
Carbon source	Maltose	HRT7.7	9.766667

Carbon source	Maltose	CONTROL	9.333333
Carbon source	Fructose	SRU4.4	12.66667
Carbon source	Fructose	SGT5.3	11.96667
Carbon source	Fructose	CONTROL	11.23333
Carbon source	Fructose	SRT9.1	11.23333
Carbon source	Fructose	HRT7.7	10.76667
Nitrogen source	Glycine	SRU4.4	11.86667
Nitrogen source	Glycine	SGT5.3	10.8
Nitrogen source	Glycine	SRT9.1	9.366667
Nitrogen source	Glycine	CONTROL	8.533333
Nitrogen source	Glycine	HRT7.7	8.466667
Nitrogen source	Yeast extract	SGT5.3	12.03333
Nitrogen source	Yeast extract	HRT7.7	11.7
Nitrogen source	Yeast extract	SRT9.1	11.7
Nitrogen source	Yeast extract	SRU4.4	11.33333
Nitrogen source	Yeast extract	CONTROL	11.16667
Nitrogen source	Tryptone	SGT5.3	13.6
Nitrogen source	Tryptone	HRT7.7	13.1
Nitrogen source	Tryptone	SRT9.1	12.93333
Nitrogen source	Tryptone	CONTROL	12.86667
Nitrogen source	Tryptone	SRU4.4	12.6
Nitrogen source	$(NH_4)_2SO_4$	HRT7.7	11.8
Nitrogen source	$(NH_4)_2SO_4$	SRU4.4	11.66667
Nitrogen source	$(NH_4)_2SO_4$	CONTROL	11.46667
Nitrogen source	$(NH_4)_2SO_4$	SRT9.1	11.2
Nitrogen source	$(NH_4)_2SO_4$	SGT5.3	10.6
Nitrogen source	NH_4Cl	CONTROL	10.76667
Nitrogen source	NH_4Cl	SRU4.4	9.933333
Nitrogen source	NH_4Cl	HRT7.7	9.366667
Nitrogen source	NH_4Cl	SRT9.1	9.166667
Nitrogen source	NH_4Cl	SGT5.3	8.7
Temperature	25°C	HRT7.7	11.83333
Temperature	25°C	SRU4.4	10.46667
Temperature	25°C	CONTROL	9.2
Temperature	25°C	SGT5.3	6.733333
Temperature	25°C	SRT9.1	5.5
Temperature	28°C	HRT7.7	15.63333
Temperature	28°C	SRU4.4	15.06667
Temperature	28°C	CONTROL	14.23333
Temperature	28°C	SGT5.3	13.6
Temperature	28°C	SRT9.1	10.1
Temperature	30°C	HRT7.7	19.8
Temperature	30°C	SRU4.4	18.96667
Temperature	30°C	CONTROL	18.9
Temperature	30°C	SGT5.3	17.2
Temperature	30°C	SRT9.1	15.66667
Temperature	35 °C	HRT7.7	18.4
Temperature	35 °C	SRU4.4	17.86667
Temperature	35 °C	CONTROL	17.2

Temperature	35 °C	SGT5.3	15.13333
Temperature	35 °C	SRT9.1	13.1
Temperature	37°C	HRT7.7	12.4
Temperature	37°C	SRU4.4	12.06667
Temperature	37°C	SGT5.3	11.7
Temperature	37°C	CONTROL	11.23333
Temperature	37°C	SRT9.1	9.666667
Starch	0,5	SGT5.3	11.13333
Starch	0,5	CONTROL	10.33333
Starch	0,5	HRT7.7	9.166667
Starch	0,5	SRU4.4	9
Starch	0,5	SRT9.1	7
Starch	1	SGT5.3	12.9
Starch	1	CONTROL	11.53333
Starch	1	SRU4.4	11.23333
Starch	1	HRT7.7	11.16667
Starch	1	SRT9.1	9.166667
Starch	1,5	SGT5.3	12.7
Starch	1,5	CONTROL	12.23333
Starch	1,5	HRT7.7	11.76667
Starch	1,5	SRU4.4	11.63333
Starch	1,5	SRT9.1	10.36667
Starch	2	SGT5.3	13.23333
Starch	2	HRT7.7	12.36667
Starch	2	CONTROL	12.3
Starch	2	SRU4.4	12.06667
Starch	2	SRT9.1	11
Starch	2,5	SGT5.3	15.06667
Starch	2,5	CONTROL	13
Starch	2,5	SRU4.4	12.96667
Starch	2,5	HRT7.7	12.93333
Starch	2,5	SRT9.1	12.26667
Starch	3	CONTROL	16.96667
Starch	3	SRU4.4	16.43333
Starch	3	HRT7.7	16.36667
Starch	3	SGT5.3	16.23333
Starch	3	SRT9.1	13.13333
Tryptone	0,5	SRT9.1	10.76667
Tryptone	0,5	HRT7.7	10.03333
Tryptone	0,5	CONTROL	9.1
Tryptone	0,5	SRU4.4	8.366667
Tryptone	0,5	SGT5.3	6.066667
Tryptone	1	SRT9.1	12.83333
Tryptone	1	CONTROL	12.2
Tryptone	1	HRT7.7	10.93333
Tryptone	1	SRU4.4	8.966667
Tryptone	1	SGT5.3	7.966667
Tryptone	1,5	SRT9.1	13.8
Tryptone	1,5	HRT7.7	13.36667

Tryptone	1,5	CONTROL	12.93333
Tryptone	1,5	SGT5.3	9.966667
Tryptone	1,5	SRU4.4	9.4
Tryptone	2	HRT7.7	14.4
Tryptone	2	CONTROL	13.9
Tryptone	2	SRU4.4	11.7
Tryptone	2	SRT9.1	9.766667
Tryptone	2	SGT5.3	6.966667
Tryptone	2,5	SRT9.1	16.26667
Tryptone	2,5	SRU4.4	16.1
Tryptone	2,5	CONTROL	15.83333
Tryptone	2,5	HRT7.7	15.83333
Tryptone	2,5	SGT5.3	11.83333
Tryptone	3	CONTROL	14.46667
Tryptone	3	HRT7.7	14.36667
Tryptone	3	SRT9.1	13.9
Tryptone	3	SRU4.4	13.03333
Tryptone	3	SGT5.3	10.8

Table S7 Specific modes of action by antagonistic bacteria against *R. solanacearum* BD 261.

Isolates	Lytic enzyme production[a]		Siderophore production[b]	Phosphate solubilization [c]
	Cellulase	protease		
Bacillus cereus strain HRT7.7	++++	+++++	++++	+++++
Paenibacillus polymyxa strain SGT5.3	++++	+++++	+++	+++++
Serratia marcescens strain SRT9.1	++++	++++	++++	++++
Enterobacter hormaechei strain SRU4.4	++++	+++++	+++	+++++

[a]Diameter of clear zone due to the production of lytic enzymes ++++ + ≥ 14 mm, ++++ ≥ 6 mm, +++ ≥ 5 mm

[b]Diameter of yellow halo on CAS agar plates ++++≥ 6 mm, +++ ≥ 5 mm

[c]Diameter of clear zones as a results of phosphate solubilisation ++++ + ≥ 12 mm, ++++ ≥ 8 mm
